# A De Novo 13q21.33-q31.1 Interstitial Deletion in a Child With Megalocornea and Neurodevelopmental Delay: A Clinico-Genomic Correlation

**DOI:** 10.7759/cureus.90646

**Published:** 2025-08-21

**Authors:** Badreddine Elmakhzen, Ayoub Nedbour, Laila Bouguenouch, Mohamed Ahakoud

**Affiliations:** 1 Medical Genetics and Onco-Genetics Laboratory, Hospital University Hassan II, Fez, MAR

**Keywords:** 13q deletion, exome sequencing, megalocornea, neurodevelopmental delay, ocular dysgenesis, pediatric genetics, pou4f1

## Abstract

Interstitial deletions of the long arm of chromosome 13 (13q) represent a rare and clinically diverse group of chromosomal abnormalities. We report the case of an eight-year-old Moroccan male with a de novo 9.8 Mb deletion at 13q21.33-q31.1, diagnosed via whole-exome sequencing (WES). The patient exhibited global developmental delay, absent structured language, macrocephaly, distinctive facial dysmorphism, ocular anomalies including bilateral megalocornea and nystagmus, musculoskeletal features such as scoliosis and hyperlaxity, and bilateral cryptorchidism. This case is the first to document megalocornea in association with a 13q deletion and supports the involvement of *POU4F1* in human corneal development.

## Introduction

Chromosome 13q deletion syndromes are clinically heterogeneous conditions characterized by a wide range of developmental and structural anomalies. The estimated prevalence is low, and many cases remain undiagnosed [1]. Interstitial deletions, A subtype of long arm of chromosome 13 (13q) deletion, represent a rare and clinically heterogeneous group of chromosomal abnormalities. The phenotypic spectrum ranges from mild developmental delay to severe syndromic presentations, including dysmorphic facial features, neurodevelopmental deficits, congenital malformations, and ocular anomalies [1]. The specific clinical manifestations depend largely on the size and location of the deleted segment and the dosage sensitivity of the genes involved [2].

Several studies have noted the importance of detailed genotype-phenotype correlation to uncover the roles of genes such as *POU4F1, DACH1*, and *EDNRB* [3,4]. These genes contribute to ocular, neural, and craniofacial development, and their deletion may lead to complex syndromic presentations, and their haploinsufficiency may underlie the clinical features observed in affected individuals [2].

Among these, deletions in the 13q21-q31 region are particularly understudied. While developmental delay, hypotonia, and macrocephaly are frequently observed, anterior segment ocular anomalies such as megalocornea have not been previously described in association with this cytogenetic abnormality [2]. 

Here, we report a unique case of an eight-year-old male with a de novo 9.8 Mb deletion at 13q21.33-q31.1 presenting with global developmental delay, dysmorphic features, and bilateral megalocornea. This case not only expands the known phenotypic spectrum of 13q deletion syndrome but also provides the first clinical evidence supporting a role for *POU4F1 *in human anterior segment development.

Interstitial deletions on chromosome 13q are associated with a variable phenotype that includes intellectual disability, craniofacial dysmorphism, and organ malformations [1]. Deletions involving 13q21.33-q31.1 are rarely described and often underdiagnosed without molecular testing. This report highlights a novel case of 13q deletion syndrome with unique anterior segment ocular manifestations and aims to expand the genotype-phenotype correlations.

## Case presentation

An eight-year-old boy was referred for evaluation of moderate global developmental delay and severe language impairment. He was born at term following a pregnancy with no documented prenatal complications. However, the parents reported signs of perinatal stress and neonatal hypotonia. Developmentally, the patient sat without support at 18 months and walked independently at four years of age. At eight years, expressive language was limited to a few isolated words. He exhibited hyperactivity, poor social engagement, and solitary play but responded appropriately to simple verbal instructions from caregivers.

Neurologically, the child had no history of seizures with the presence of ataxic symptoms. An electroencephalogram (EEG) demonstrated a symmetric, well-organized background with some slow-wave activity, without any epileptiform discharges.

Physical examination revealed a distinctive craniofacial dysmorphism, including macrocephaly, hypertelorism, and a flat nasal bridge. Ocular assessment showed bilateral megalocornea, blue irides, right divergent strabismus accompanied by nystagmus jerks, and amblyopia. The photomotor reflex is present. Refractive correction has been prescribed with a spherical equivalent of -3.00 for the right eye and -0.25 for the left eye. Examination revealed an iris pigmentation abnormality with a sapphire blue hue. Fundoscopic evaluation showed a small, hypoplastic optic disc with chorioretinal atrophy in the right eye, while the left eye exhibited a normal fundus. He had no cataract or lenticular dislocation reported.

Musculoskeletal findings included moderate scoliosis and generalized ligamentous hyperlaxity. Genital examination and abdominal ultrasound revealed bilateral cryptorchidism. Cardiac echocardiography and hearing tests were normal.

Whole-exome sequencing (WES) was initially performed, which revealed a heterozygous pathogenic copy number loss in 13q chromosome described as: NC_000013.11:g.(?_69707625)_(79543682_?)del (GRCh38).

This 9.8 Mb deletion on chromosome 13q21.33-q31.1 includes 26 protein-coding genes ( OMIM genes are in bold), namely:***KLHL1**, DACH1, MZT1, BORA, DIS3, **PIBF1**, KLF5, KLF12, **TBC1D4**, COMMD6, UCHL3, LMO7DN, KCTD12, ACOD1, **CLN5, FBXL3,** MYCBP2, SCEL, LOC100129307, SLAMF1, **EDNRB, POU4F1**, OBFC1, RBM26, NDFIP2.*

This copy-number variant is not observed in the gnomAD SVs v2.1.1 dataset and overlaps with previously reported pathogenic deletions [5]. Supporting data from ClinVar (VCV0001495782) [6], DECIPHER (401244) [7], and Orphanet (ORPHA:262101) [8] link deletions in this region with neurodevelopmental syndromes, ocular anomalies, and dysmorphism. The region is subject to haploinsufficiency. Based on these findings, the deletion was classified as pathogenic.

In light of these findings, confirmatory orthogonal testing such as chromosomal microarray (CMA) or whole-genome sequencing (WGS) may be considered to validate breakpoints and assess for additional genomic imbalance.

**Figure 1:**
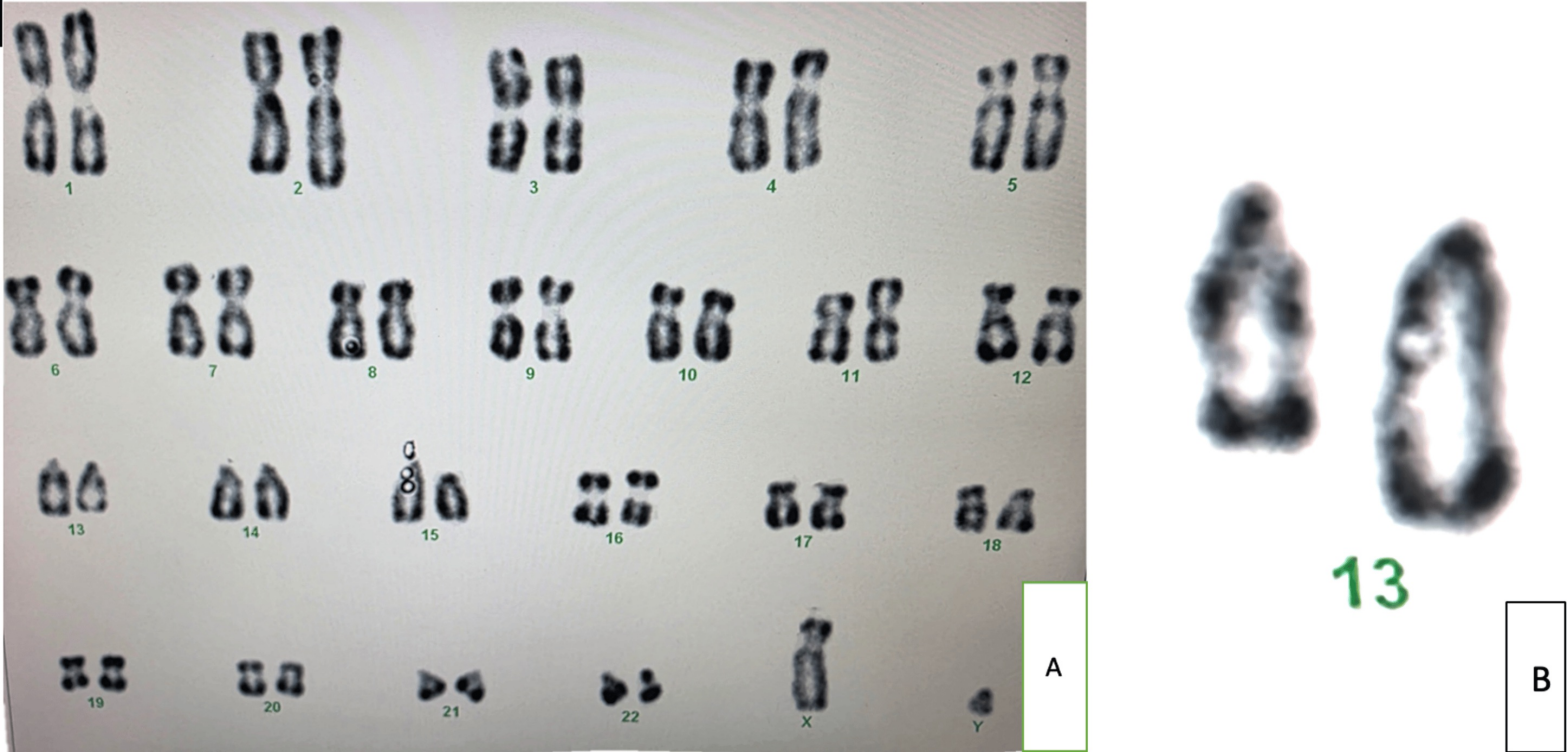
A: R-banded karyotype showing a normal XY chromosomal complement with a deletion on the long arm (13q) of the right chromosome 13. B: Interstitial 13q deletion on the left chromosome 13, with its normal homolog on the right

CGH testing was not performed due to financial constraints and limited accessibility in the local clinical setting. We opted instead for standard karyotyping, which revealed a deletion on chromosome 13 (Figure 1). This deletion was initially missed on the first karyotype and was later detected by WES. WES identified a heterozygous de novo 9.8 Mb deletion spanning 13q21.33 to 13q31.1. Parental testing confirmed the deletion as de novo, with both parents showing normal karyotypes, which significantly reduces the recurrence risk-although gonadal mosaicism cannot be excluded.

The deleted segment contains 26 protein-coding genes. Notable among these are the following genes: *POU4F1*, which is associated with retinal ganglion cell (RGC) development; mouse models show disrupted corneal morphogenesis in its absence, and our patient’s megalocornea aligns with this phenotype. *EDNRB* encodes the endothelin B receptor; homozygous mutations cause Waardenburg syndrome type IV, while haploinsufficiency may lead to isolated iris hypopigmentation. *DACH1* is a transcription factor essential for ocular, neural, and limb development, and has been associated with intellectual disability and macrocephaly. *DIS3* is a component of the RNA exosome complex and is crucial for neuronal development.

A thorough multidisciplinary care plan has been established to meet the patient's complicated medical and developmental needs. In order to manage amblyopia and screen for cataracts, routine ophthalmologic evaluations are carried out. Scoliosis monitoring accompanied by physiotherapy therapies is part of orthopedic follow-up. Speech and psychomotor therapy have been undertaken to boost language development and motor coordination. The patient has been sent to a urologist and an endocrinologist for a hormonal assessment and surgery to treat cryptorchidism. Enrollment in a special education program, with a customized assistance plan based on behavioral and cognitive needs, provides educational help. Genetic counseling was presented to the parents, including advice for prenatal diagnosis in future pregnancies to assess recurrence risk.

Overall, the patient presents with moderate global developmental delay, severe language impairment, ataxia, and behavioral features including hyperactivity and poor social interaction. Distinctive craniofacial dysmorphism (macrocephaly, hypertelorism, and flat nasal bridge) is accompanied by ocular anomalies, bilateral megalocornea, blue irides, divergent strabismus, nystagmus, and amblyopia, along with a hypoplastic optic disc and chorioretinal atrophy in one eye. Additional findings include generalized ligamentous hyperlaxity, moderate scoliosis, and bilateral cryptorchidism, with otherwise normal cardiac and auditory assessments.

## Discussion

Several of the genes within the deleted region are known or predicted to be associated with autosomal dominant (AD) inheritance, which is particularly relevant for understanding the phenotypic outcome in this case. These include *POU4F1*, associated with childhood-onset ataxia and hypotonia syndrome (OMIM #619352); *FBXL3*, associated with intellectual developmental disorder with short stature, facial anomalies, and speech defects (OMIM #606220); and *EDNRB*, which can cause Waardenburg syndrome type IV in both dominant and recessive contexts (OMIM #277580). Other candidate AD genes include *DACH1, KCTD12, KLF5*, and *RBM26*, all of which have been implicated in syndromic developmental delay, neuropsychiatric traits, or cancer predisposition based on case reports or CNV analyses (Table 1) [9]. The deletion of these dosage-sensitive genes may contribute additively to the observed neurodevelopmental and systemic manifestations in this patient [1].

**Table 1:** Ad genes deleted in 13q21.33-q31.1 and their associated conditions reported in OMIM *Indicates that it had two phenotypes for the second clinical form. ?Indicates the clinical implication as an autosomal dominant (AD) condition is contradictory. AD: autosomal dominant; N/A: not applicable

Gene	OMIM ID	Associated condition	Inheritance
POU4F1	619352	Ataxia, intention tremor, and hypotonia syndrome	AD
DIS3	607533	N/A	AD
FBXL3	606220	Familial advanced sleep-phase syndrome 2	AD
EDNRB	277580/600155	Waardenburg syndrome type IV/{Hirschsprung disease, susceptibility to, 2}	AD*
DACH1	N/A	Candidate in syndromic developmental delay	AD?
KCTD12	N/A	Candidate neurodevelopmental disorder	AD?
KLF5	N/A	Candidate in cancer predisposition	AD?
RBM26	N/A	Possible role in epilepsy	AD?

13q deletion syndrome encompasses a spectrum of chromosomal abnormalities involving terminal or interstitial losses along the long arm of chromosome 13, with clinical manifestations that vary significantly depending on the location and extent of the deleted segment [3]. The syndrome is typically classified into three major forms based on deletion position: proximal (13q12-13q21), intermediate (13q21-q31), and distal (13q31-q34). Each subtype exhibits distinct clinical characteristics. Proximal deletions often result in severe brain malformations, holoprosencephaly, and profound developmental delay. Intermediate deletions, such as in our case (13q21.33-q31.1), are associated with intellectual disability, hypotonia, macrocephaly, and occasionally, subtle ocular or skeletal anomalies. Distal deletions encompassing 13q33-q34 are more frequently associated with congenital heart defects, genitourinary malformations, and seizures [4]. Importantly, genotype-phenotype correlations remain challenging due to the variable expressivity and incomplete penetrance often observed across cases.

One particularly novel aspect of our case is the anterior segment ocular anomaly, specifically, bilateral megalocornea, that could possibly be linked to the deletion of the *POU4F1* gene. Although* POU4F1 *(also known as *BRN3A*) has been extensively studied for its role in RGC differentiation, its involvement in corneal and anterior segment development in humans had not been previously documented. In a foundational murine study, *POU4F1*-deficient mice demonstrated significant anterior segment abnormalities, including reduced stromal thickness, deepened anterior chambers, flattened corneal curvature, and disrupted collagen alignment, largely attributed to impaired TGF-β signaling [2,10]. Our patient's ocular phenotype represents the first clinical correlation supporting the hypothesis that *POU4F1* may play a dosage-sensitive role in human anterior segment morphogenesis.

Beyond its ocular role, *POU4F1* has mainly been associated with neurological manifestations, including childhood-onset ataxia, intention tremor, and axial hypotonia. This expanded phenotype is consistent with the gene's expression in cerebellar and brainstem motor pathways. According to OMIM and curated GenCC data, alterations in* POU4F1* may result in a syndrome marked by neurodevelopmental delay, motor incoordination, and cerebellar-like symptoms [11]. In our patient, the presence of early axial hypotonia, delayed gross motor milestones, and poor coordination with ataxia is compatible with this phenotype [12]. This case further supports the role of *POU4F1* haploinsufficiency in both anterior segment dysgenesis and neuromotor developmental delay.

Further support for the role of POU4F1 in motor development comes from the description of a cohort of patients with de novo heterozygous loss-of-function mutations in *POU4F1* [13]. Affected individuals exhibited a consistent phenotype characterized by early-onset axial hypotonia, gait ataxia, intention tremor, and delayed gross motor milestones, alongside variable cognitive deficits. Neuroimaging showed mild cerebellar vermis hypoplasia in some cases. Notably, several patients also exhibited visual tracking abnormalities, raising the possibility of *POU4F1* involvement in both the anterior visual pathway [13]. These findings strongly support the hypothesis that the gene haploinsufficiency affects both ocular and cerebellar systems, correlating well with the phenotype observed in our patient. This convergence of evidence strengthens the inclusion of *POU4F1* in gene panels for syndromic developmental delay with ocular and neuromotor features.

Beyond motor and anterior segment involvement, *POU4F1* also plays a critical role in RGC development, as demonstrated by studies showing that the POU4F1-Tbr1 transcriptional cascade is essential for the specification of a subset of RGC that express the cell adhesion molecule Jam2 [11]. This population of RGCs contributes to retinotopic mapping and synaptic organization. Disruption of this cascade in murine models results in retinal disorganization, aberrant axonal targeting, and defective visual circuits. Although our patient’s visual impairment was primarily attributed to anterior segment anomalies, the presence of nystagmus and amblyopia may also reflect underlying retinal circuitry deficits due to *POU4F1* haploinsufficiency. These findings reinforce the multifunctional role of *POU4F1* across multiple compartments of the visual system.

## Conclusions

This case illustrates the diagnostic value of genomic tools such as WES in detecting clinically significant copy-number variants that may be missed by conventional karyotyping. It underscores the importance of detailed phenotype-genotype correlation in identifying novel clinical features associated with known chromosomal deletion syndromes. The association between 13q21.33-q31.1 deletion and bilateral megalocornea suggests an emerging ocular phenotype deserving of further exploration. Close developmental follow-up and early therapeutic interventions are crucial to optimizing functional outcomes in children with rare genomic deletions.

The observed 13q21.33-q31.1 interstitial deletion, encompassing key developmental genes including *POU4F1, DACH1, and EDNRB*, is associated in this patient with a constellation of features: moderate developmental delay, severe language impairment, bilateral megalocornea, and distinct craniofacial and ocular abnormalities. Notably, the presence of bilateral megalocornea, not previously reported in association with this specific cytogenetic region, suggests a potentially novel ocular phenotype. The involvement of *POU4F1* raises a hypothetical link between this gene and the observed ocular findings. However, further studies are needed to confirm this association in humans. These findings expand the phenotypic spectrum of 13q deletion syndromes and advocate for WES or WGS as first-tier diagnostic tools in children with unexplained developmental delay and syndromic features. Ongoing multidisciplinary management remains essential to address medical, developmental, and educational needs while monitoring for emerging complications.
